# First Report of *Acanthocheilonema reconditum* Outbreak in Canines with Clinical Signs of Anemia from Southwestern Colombia

**DOI:** 10.3390/pathogens11121434

**Published:** 2022-11-28

**Authors:** Nathaly Espinosa, Angelo Rosero, Claudia Lucia Villegas, Isabel Cristina Garcia, Tania Gaviria-Cantin, Alejandra Peña Nieto, Beatriz E. Ferro, Luisa Maria Nieto Ramirez

**Affiliations:** 1 Facultad de Ciencias Básicas, Universidad Santiago de Cali, Santiago de Cali 760035, Colombia; 2 Laboratorio Clínico Veterinario Zoolavet, Santiago de Cali 760033, Colombia; 3 Departamento Administrativo de Planeación Municipal, Santiago de Cali 760045, Colombia; 4 Facultad de Ciencias de la Salud, Universidad Icesi, Santiago de Cali 760031, Colombia

**Keywords:** filariasis, dogs, microfilariae, hemoglobin, *Dipetalonema reconditum*, PCR–RFLP, Cali, zoonosis

## Abstract

Different nematodes affect canines, however *Acanthocheilonema reconditum* was considered mostly a non-pathogenic parasite. Climate change, animal migration, and other factors transformed the dynamics of vector-borne diseases, including filariasis. Since 2016, a sudden increase in the number of dogs with microfilaremia was reported by different veterinary centers in Cali, southwest Colombia. The objective of this study was to molecularly identify the etiologic agent of this filariasis outbreak detected in this city, using PCR–RFLP and evaluating dogs’ clinical signs. From 2018–2019, canine filariasis cases were prospectively evaluated after a microscopic test, recruiting 82 cases and 43 healthy controls from 2971 samples. *Acanthocheilonema reconditum* (Nematoda, Onchocercidae) was identified in 61.3% of the cases (49/82) by PCR–RFLP. Sanger sequencing of the 5.8S ribosomal RNA gene and internal transcribed spacer-2 fragment was additionally performed on seven cases, confirming *A. reconditum* in all of them. The filariasis cases are statistically associated with male dogs who have clinical signs of anemia, low levels of hemoglobin and hematocrit (*p* < 0.0001), and high levels of plasma proteins (*p* < 0.001). This emerging canine disease constitutes an important public health concern among veterinarians and active surveillance is advised to explore its zoonotic potential.

## 1. Introduction

Filariasis is a parasitic disease caused by nematodes of the family Onchocercidae that affect a wide variety of mammals. Microfilariae is the first larval stage of the nematode that can be observed in peripheral blood, representing the parasite diagnostic stage [1,2]. Depending on the geographical area, the most commonly observed filariae in dogs is either *Dirofilaria immitis*, the causing agent of heartworm disease, observed in the Americas [1,3], or *D. repens* that is observed particularly in European countries, causing subcutaneous nodules, mostly asymptomatic [4]. However, there is another filariae species that affects canines, *Acanthocheilonema reconditum* (previously known as *Dipetalonema reconditum*), which can be found subcutaneously, causing a range of symptoms from no clinical signs to skin and soft tissue-related pathologies and whose frequency has been increasing recently [5,6].

Canine filariasis has been detected in South America since 1875, mainly caused by *D. immitis.* Countries such as Brazil, Argentina, and Peru reported this disease with different frequencies (from 3.5 up to 23.5%) in the last two decades [7,8]. The distribution of filariasis in Colombia has been mainly determined using microscopic and serological tests that detect the *D. immitis* antigen, with a frequency ranging from less than 0.5% in the Andean to 20% in the Caribbean region in the last 18 years [2,7,8]. The last study published from southwest Colombia in 1967 showed a prevalence of 5% for *D. immitis* in canines from Cali, the city with the largest metropolitan area [2,9]. However, there are no recent studies about the distribution of filarial nematodes among dogs in southwest Colombia. *Acanthocheilonema reconditum*, on the other hand, has been mainly reported in canine filariasis cases from Brazil [10,11] and some countries in Central America [12,13,14].

In 2016, veterinary diagnostic centers of Cali, Colombia detected an unusually high number of dogs with microfilariasis in the bloodstream compared to previous years, suggesting a canine filariasis outbreak in the southwestern region of this country. Interestingly, most cases (detected by microscopy) were negative for commercial serologic tests for *D. immitis*, generating concerns about the etiology of the cases observed. Unfortunately, there are no official records regarding the prevalence of this parasitic disease or potential zoonotic cases in Colombia. All these facts emphasize the need for novel and more specific diagnostic approaches to identify the filarial species, which can help to establish better surveillance measures and explore potential clinical signs among the affected animals. For this reason, here we conducted a prospective study that aims to identify, by PCR–RFLP, the circulating filariae in canines from southwest Colombia and to characterize the clinical signs of the cases registered for a one year period. 

## 2. Results

### 2.1. Microscopic and Serologic Test

**Figure 1:**
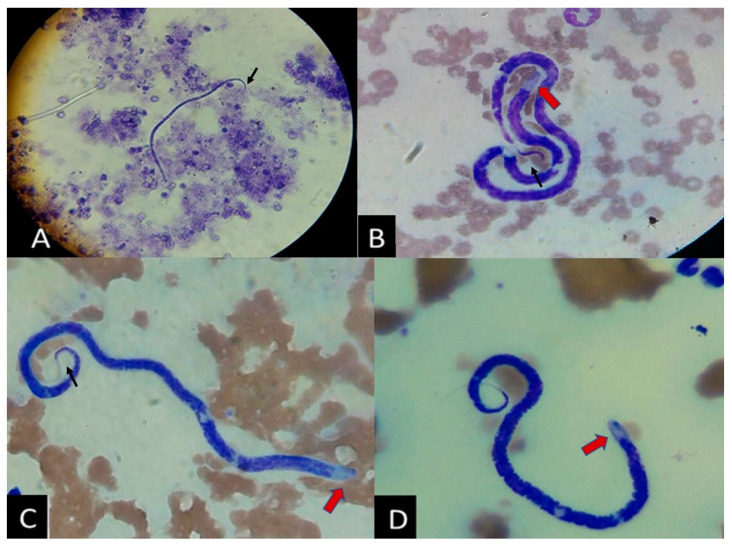
Microscopic detection of microfilariae in dogs from southwestern Colombia. (**A**) Knott’s test (observed in 40×). (**B**–**D**) Wright-stained blood smears of three representative cases, showing microfilaria features (100×). Red and black arrows indicate features of the parasite’s heads and tails respectively.

From August 2018 to August 2019, 2971 blood samples from different canines were analyzed for the presence of microfilariae. A total of 102 (3.4%) dogs are identified with microfilariae by microscopic examination, but only 82 dog owners gave their consent to participate in the study. The microscopic identifications of the cases include the observation of microfilariae with a curved tail (black arrows) and a clear-blunt head (red arrows) (Figure 1), which are typical features of *A. reconditum*. Only two cases (cases No. 30 and 46) are positive for the *D. immitis* antigen. Moreover, 43 healthy individuals are included as controls with negative results for both microscopic and serologic tests.

### 2.2. Characteristics of the Study Population

The median age for both canine filariae cases and healthy controls is 5 years, with an interquartile range (IQR) of 2–9 years for cases and 3–8 years for controls. Canine filariasis cases are mostly male (62/82), from the mestizo/mixed breed (46/82), followed by the Poodle and Schnauzer breeds (Table 1 and Table S1). 

### 2.3. PCR-RFLP and Sanger Sequencing 

Out of the 82 microscopically identified filaria cases enrolled in the study, 68.3% (56/82) are positive for the amplification of the 5.8S–ITS2–28 region using the PCR primers targeting ribosomal subunits of different filariae. The PCR was also performed for the 43 controls, all of which are negative. The latter results give us a sensitivity of 68.3% and specificity of 100% for the PCR test. 

RFLP analysis of the PCR product allows for the molecular identification of 49 *A. reconditum* cases among the 56 PCR positive cases. The positivity is determined by the presence of one band of about 290 bp in the agarose gel, representing two DNA fragments of 285 and 288 bp after the enzymatic digestion. 

**Figure 2:**
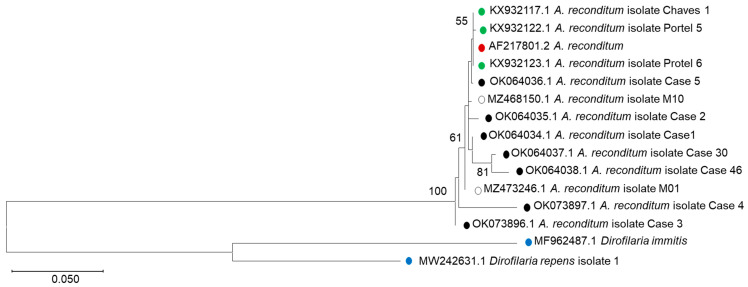
Phylogenetic analysis of the ribosomal DNA inter-spacer (5.8S–ITS2–28S) sequences by the maximum likelihood method and Tamura 3-parameter model (21). The analysis was conducted in MEGA X (20). Filaria cases from this study are depicted in black circles, other cases reported from Colombia (isolates M1 and M10) in white, and cases from South America (Brazil) in green circles. *Acanthocheilonema reconditum* reference strain is shown in red circle. *Dirofilaria immitis* and *D. repens* are used as outgroups (blue circles). Bootstrap (≥50) from 1000 replications appears next to the corresponding branch. Bar, 0.05 substitutions per nucleotide position.

Sanger sequencing of the PCR products of the first five cases (1–5, GenBank accession numbers OK064034, OK064035, OK073896, OK073897, and OK064036) confirms our PCR–RFLP results, identifying *A. reconditum* with a percentage identity close to 100% in most of the cases. Additionally, cases 30 and 46, which exhibit a positive test for the detection of *D. immitis* antigen, are also positive for *A. reconditum* according to the PCR–RFLP test. To solve this discrepancy, Sanger sequencing was performed for both cases, identifying these as *A. reconditum* with an identity of 99.4% for case 30 (OK064037) and 98.6% for case 46 (OK064038), according to BLAST search. Phylogenetic analysis was performed including some other isolates reported from Colombia (white circles, Figure 2) and from the South American region (green circles, Figure 2). As expected, the isolates found in this study (black circles, Figure 2) cluster with the other *A. reconditum* found in Colombia (isolates M01 and M10). However, there is a close relationship with the isolates identified in Brazil (green circles, especially case 5, Figure 2).

### 2.4. Hematological Findings and Clinical Signs 

Hematocrit and hemoglobin are significantly reduced in all filaria cases compared to controls (*p* < 0.0001). Furthermore, a significant increase in the plasmatic protein levels is found for all cases (*p* = 0.0015) with a median of 79 g/L, which is above the reference values (60–75 g/L). On the other hand, there is not a significant difference in the eosinophil count or any other blood cell population between filaria cases and controls (Table 2). Regarding clinical characteristics, infected dogs have clinical signs of anemia (such as weakness, 12/82), followed by skin problems (6/82), bleeding (including ulcers and eye bleeding, 6/82), malnutrition and cachexia (5/82), and respiratory distress (5/82). However, half of the filaria cases do not reveal evident signs of disease (40/82). Interestingly, about 39% (32/82) of owners report their dogs having infestation with one or more types of ectoparasites (fleas, ticks, or lice). 

A bivariate analysis reveals that dogs that test positive for *A. reconditum* have an increase in the likelihood of having anemia (hemoglobin < 11.9 g/dL) by 16.3 times (Table 3). 

### 2.5. Geographic Distribution of Filaria Cases

**Figure 3:**
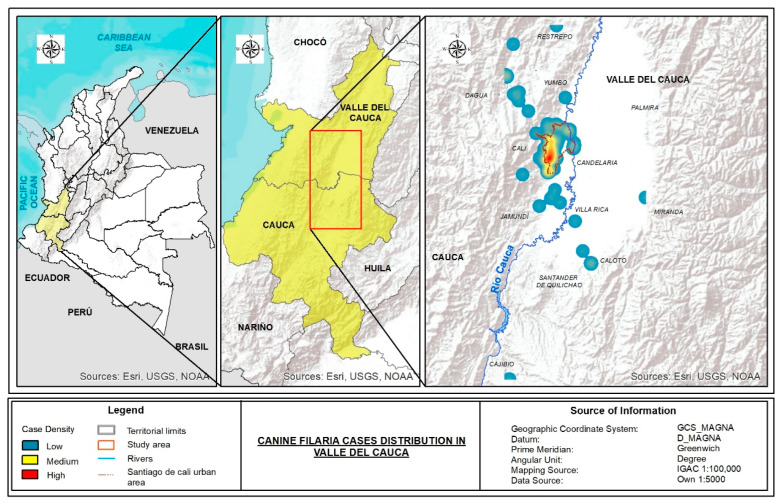
Geographical distribution of the canine filaria cases detected in southwest Colombia. ESRI: Environmental Systems Research Institute, USGS: United States Geological Survey, NOOA: National Oceanic and Atmospheric Administration.

The canine filariasis cases are largely distributed in the urban area of Cali (represented as red dotted lines in right panel of Figure 3), specifically, in southwestern neighborhoods (34/82, 41.5%). On the other hand, around 13.4% (11/82) of the filariasis cases are from neighboring cities of the state of Valle del Cauca (such as Restrepo, Dagua, Yumbo, and Jamundí), and 6.1% from northern rural areas from the state of Cauca (Figure 3). 

## 3. Discussion

The presence of microfilariae in dogs from southwestern Colombia was investigated for one year, detecting 102 positive cases by microscopic tests, with 82 of them further analyzed by molecular tests. This unusually high number of canine filariasis detected in Cali and close cities represents an outbreak. It is important to emphasize that filariasis is not part of current public health surveillance in Colombia. 

Although *A. reconditum* has a worldwide distribution [5,11,17,18,19,20,21,22,23,24,25,26,27], the high frequency of this species in southwestern Colombia is unprecedented, since *D. immitis* has been the most frequently reported filaria species among canines from Colombia and South America [2,8,9,28,29]. A recent study in Brazil also identified a higher distribution of *A. reconditum* over *D. immitis* (7.2% versus 2.2% in 418 samples tested) [11]. Hence, the high number of *A. reconditum* observed in southwestern Colombia is not an isolated event in the continent and underscores the need for expanding epidemiological surveillance to additional vector-borne parasites.

In relation to the molecular test performed, about 68.3% (56/82) of dogs test positive via PCR, and 85.7% of the PCR-positive cases (48/56) are confirmed to be infected with *A. reconditum* by RFLP. Filaria cases that are PCR-negative/microscopic-positive (31.7%, 26/82) could be attributed to the low parasitemia at the sampling time. This low parasitemia could be explained by two factors: first, the circadian rhythm of the parasite [30], affecting the parasite´s DNA content in the dog’s bloodstream, or second, by the intake of non-reported medication between the initial diagnosis stage and the time of sampling for DNA isolation (although this specific information was not asked the canine’s owner). It is important to note that once the dog tested positive by the microscopic test, the owner was invited to bring his/her dog to be enrolled in this study for molecular and epidemiological characterization. Therefore, although the sample corresponded to the same animal, it was taken at different times for both microscopic and molecular tests. We also acknowledge the PCR–RFLP method is very sensitive to DNA concentration and, for future attempts, this should be a consideration. For instance, first standardize a minimum level of parasitemia, since DNA concentration can be affected by the DNA content of host cells. 

We detect a discrepancy between the serological and PCR–RFLP tests for two cases (cases No. 30 and 46), that could be solved by the PCR sequencing analysis. As observed in Figure 2, cases No. 30 and 46 are identified as *A. reconditum*. Although the antigen test applied in the study has a sensitivity of 94% and specificity of 100% (according to the manufacturer), the discrepant results may be due to cross-reactivity with *A. reconditum* antigens. Cross-reactivity between *D. immitis* and *Acanthocheilonema* species such as *A. reconditum* and *A. odendhali* was previously described [30,31]. Additionally, shared epitopes are reported between *D. immitis* and other nematode species such as *Toxocara canis* and *Angiostrongylus vasorum* [30,32,33,34]. Our findings underline the need for new biomarkers for rapid diagnostic tests that could detect not only *D. immitis*, but also other filarial species.

*A. reconditum* is known for its low pathogenicity in canines and association with mild clinical manifestations in the subcutaneous tissue and skin [6,35]. However, this study is the first report of a significant association between the presence of this parasite and anemia in dogs (Table 3). Additionally, the high levels of plasmatic proteins found in the filariasis cases suggest an inflammatory response in these dogs (Table 2). A reduction in hemoglobin concentration and an increase in total protein levels was previously reported among *A. reconditum* cases, attributing those findings to the destruction of erythrocytes and the increase in antibody response to parasitic antigens [36]. 

Interestingly, 78% of the microscopic positive cases for filariasis are male (64/82). In fact, our study finds that the risk of acquiring filariae infection is about nine times higher in male than female dogs (*p* < 0.00001, Table 1). Two previous studies carried out in Colombia did not find a significant relationship between male dogs and microfilariae infection [28]. In line with our results, a study carried out in Vesuvius, Italy, between May 1999 and June 2000 shows that male dogs have a significantly higher risk for *A. reconditum* infection [37]. A higher prevalence of *A. reconditum* in male canines (85.7%) is proposed to be attributed to hormonal effects [36].

We observe a higher concentration of cases in the southwest of Cali (Figure 3). This cluster of canine filariasis coincides with the location of the largest dog refugee centers, as well as recent informal and formal urbanization projects. This area of current development has a greater distribution of vegetation (compared with the other parts of the city), with small water bodies (such as Melendez, Lili, and Pance rivers and small ponds, Figure S1). Additionally, according to the local environmental administration reports, the southwest of Cali has a higher distribution of urban wetlands and has the lowest surface temperatures in the urban area of the city (ranging from 17.4–23.8 °C, Figure S1) [38,39]. These environmental conditions may favor the growth and dissemination of different vectors for filariae. It is necessary to explore the presence of previously known (fleas and lice) and potentially new vectors for *A. reconditum* in the city that could affect the geographic distribution of this filariae. 

We acknowledge some limitations of our study, such as the need to expand the PCR sequencing analysis to more samples, however, the number of sequenced strains was constrained by the budget restrictions. Additionally, we did not quantify the parasitemia at the time of molecular testing, which could have helped to explain the reason for the low positivity rate of the PCR test (70%, 56/82). It is important to conduct a prospective study that can help to describe the clinical outcome of *A. reconditum* infections in canines, especially those cases that develop anemia, to further evaluate potential treatment options.

## 4. Materials and Methods

### 4.1. Sample Collection and Study Setting

Positive canine filariasis cases detected by microscopy from August 2018 to August 2019 were selected for this prospective study. The canines were initially diagnosed at a veterinary center in Cali, Colombia. The animals’ owners were asked to bring the animal to enroll in the study, provide a new blood sample, and fill out an informed consent before the canines were included in the study. In parallel, samples from healthy dogs of different sex and breeds were collected as a control group. 

### 4.2. Variables

A case report format (CRF) was filled out including the clinical and other characteristics such as the canine age, breed, address during the last year, presence of ectoparasites (lice, fleas, and/or ticks), and any noticeable clinical sign such as weakness, malnutrition or cachexia, skin lesions, bleeding, ulcers, respiratory distress, seizures, among others. Participants were identified with a code to ensure data confidentiality.

### 4.3. Clinical Laboratory Testing

In the initial diagnosis stage, blood smears were stained with Wright and a thick drop stained with Giemsa or Knott´s test for a microscopic examination that allowed us to recruit positive cases. Blood was collected by venipuncture and mixed with ethylenediaminetetraacetic acid (EDTA, as an anticoagulant) for both cases and control groups. All cases detected by microscopy were also analyzed with a serological test that specifically detects the secreted 14KDa antigen of *D. immitis* [40] (Canine Heartworm Ag 2.0 Bionote, Inc., Hwaseong-si, Korea). These microscopic and serological tests were also performed for the control group. Additionally, a comprehensive hemogram examination was performed for cases and controls, which included the determination of hemoglobin, hematocrit, leukocytes, and red blood cell counts, using the KT3800 Vet Auto Hematology analyzer (Kindle, Beijing, China). Blood cell population and morphological features were confirmed by blood smear microscopic analysis by trained laboratory personnel. Total plasmatic proteins were analyzed using a refractometer.

### 4.4. Molecular Tests 

#### 4.4.1. DNA Extraction

After the positive cases and controls were enrolled in the study, a sample of venous blood was collected with EDTA-coated tubes using 200 µL of anticoagulated blood from positive cases (previously detected by microscopy) and controls, using the commercial kit (Thermo Scientific™ GeneJET ™ DNA Purification Kit, Shanghai, China), following the manufacturer’s instructions. 

#### 4.4.2. Polymerase Chain Reaction (PCR) 

We conducted a PCR amplification of the 5.8S–ITS2–28S ribosomal subunits of different filariae, using the pan-filarial primer pair as well as the cycling conditions reported by Rishniw et al., 2006 [41]. Briefly, PCR mixture contained 1.5 mM MgCl_2_, 0.25 mM dNTPs (Promega, Madison, WI, USA), 0.5 µM each primer DIDR-F1 and DIDR-R1 (Macrogen), 0.05 U/µL *Taq* polymerase, and 1X *Taq* buffer (Promega), in a final volume of 20 µL. About 50 ng/µL of DNA was added to the mixture. After the cycling conditions, PCR products were analyzed in 1.5% agarose gels and stained with SYBR^®^ safe (Merck, Shanghai, China). DNA of three filariae species (*D. immitis*, *A. reconditum* and *D. repens*) donated by Università Degli Studi di Bari in Valenzano, Italy and Universidad de la Plata, Argentina, were used as positive controls to validate our molecular tests.

#### 4.4.3. Restriction Fragment Length Polymorphisms (RFLP)

We used the in-silico-predicted sequence of the PCR products for the three most reported filaria species among dogs (*D. immitis, A. reconditum,* and *D. repens*), using the parasite sequences, the pan-filarial primer pair defined previously by Rishniw et al. [41], and the software SnapGene^®^ version 5.1.5. With these sequences, we identified the restriction enzyme *Mwo*I that selectively generates two products of 285 and 288 base pairs (bp) from the *A. reconditum* amplicon, without cutting the PCR product obtained from the other two filariae species (*D. repens* and *D. immitis*). RFLP was then performed in a final volume of 15 µL, which contained 1.5 µL of ultrapure water, 1.2 µL of enzyme buffer, 0.3 µL of the enzyme (*Mwo*I), and 12 µL of product from the PCR test. The enzymatic digestion mixture was incubated at 60 °C for 15 min, following manufacturer’s instructions. The digestion products were visualized in 1.5% agarose gels. 

#### 4.4.4. Sanger Sequencing of the PCR Products

Sanger sequencing of the 5.8S–ITS2–28S ribosomal DNA amplicons was performed for eight of the samples using the 3500 Genetic Analyzer and the BigDye™ Terminator v3.1 Cycle Sequencing Kit (ThermoFisher Scientific) at the sequencing core of Universidad Icesi. DNA sequences obtained with forward and reverse primers were trimmed using the software BioEdit version 7.2.5. Then, trimmed sequences were aligned using the muscle algorithm in MEGA X version 10.1.7 and the final consensus sequence for each strain was then created in BioEdit version 7.2.5. Consensus sequences were analyzed basic local alignment search tool (BLAST) and submitted to nucleotide database GenBank from https://www.ncbi.nlm.nih.gov/ (accessed on 13 September 2021). MEGA X was also used to build a maximum parsimony (ML) phylogenetic tree with 1000 iterations [42].

### 4.5. Georeferencing of Cases

All canine filariae cases were geocoded, consolidating all the addresses in an alphanumeric format in Microsoft Excel. Then, the coordinates were assigned using Google Maps and OpenStreetMap. We used ArcGis 10.2 software to assign the coordinates and the point of each address, consolidating a graphical output with a base map of the city and its surroundings (source Instituto Geográfico Agustín Codazzi of Colombia). Additionally, a heatmap was built by the transformation of vector files to raster, using a colored grid to represent clusters. A traffic light color scale was assigned, where red represents the higher concentration of cases.

### 4.6. Statistical Analysis

For continuous variables, a Shapiro–Wilk normality test was performed and the medians between the groups were compared using either an unpaired *t*-test or Mann–Whitney depending on data distribution. Bivariate analysis was applied to evaluate the association between anemia (hemoglobin < 11.9 g/dL) with the *A. reconditum* RFLP-confirmed cases compared to the control group. Statistical analysis was performed using Graph Pad Prism software (version 8.4.2) and STATA/MP 14.0 for Windows. Sensitivity and specificity for the PCR test compared with the current microscopic gold standard were calculated using OpenEpi version 3 (https://www.openepi.com/, accessed on 24 January 2022). 

### 4.7. Ethical Considerations

All the procedures described in this work had the approval of the Institutional Ethics Committee for Care and Animal Use in Experimentation (CIECUAE) of the Universidad Icesi, Cali, Colombia, filed under the number 0019/2018. Blood samples from canines were taken from trained personnel, minimizing their discomfort. Canine owners approved the participation of their pets in the study by signing the informed consent document. 

## 5. Conclusions

Our pioneering study evaluating this parasitic disease in Colombia by microscopic test combined with the PCR–RFLP test using the enzyme *Mwo*I allowed for the detection of *A. reconditum* circulating in dogs in the southwestern Colombia. This study led us to identify *A. reconditum* as the most frequent causing species of the canine filariasis outbreak in Cali during 2018–2019. This is the first time that this nematode species is reported in such a high frequency in Colombia and, more importantly, associated with hematological signs of anemia. Although *A. reconditum* is not known as a highly pathogenic species, this study highlights the importance of including it in the differential diagnosis of anemias in canines from Colombia, and potentially worldwide. It is possible that climate change, together with animal migration and commercial trades, provide the conditions that allow the proliferation of vectors that transmit this nematode. At the public health level, it is necessary to alert the medical community about this canine pathogen, since it has the potential to infect people, as demonstrated in a human case of *A. reconditum* already reported in Australia, causing eye discomfort and irritation [43].

## Figures and Tables

**Table 1:** Characteristics of the analyzed dogs enrolled in the study.

Variable	Microscopic Positive Cases	Controls	Odds Ratio (95% CI)	*p*-Value ^1^
	*n* (%)	*n* (%)		
*Age groups (years)*				0.57
≤1.5	13 (15.9)	8 (18.6)	1	
1.5–8	49 (59.8)	28 (65.1)	1.1 (0.4–2.9)	
≥8.1	20 (24.3)	7(16.3)	1.7 (0.5–5.7)	
*Sex*				<0.0001
Female	18 (22)	31 (72.1)	1	
Male	64 (78)	12 (27.9)	8.9 (3.8–20.8)	
*Dog’s breed*				0.18 ^2^
Other	11 (13.4)	10 (23.3)	1	
Schnauzer	7 (8.5)	6 (14.0)	1.1 (0.3–4.2)	
Labrador retriever	5 (6.1)	2 (4.6)	2.3 (0.4–14.5)	
Miniature Pinscher	3 (3.7)	2 (4.6)	1.4 (0.2–9.9)	
Mestizo/Mixed	46 (58.5)	23 (53.5)	1.8 (0.7–4.9)	
Poodle	8 (9.8)	0 (0)	-	

^1^ Pearson chi-square. ^2^ Fisher exact test. CI: confidence intervals.

**Table 2:** Comparison of hemogram results between filaria cases and controls.

Variable	Units	Reference Values ^1^	Cases (Median)	Controls (Median)	*p*-Value ^2^
Hemoglobin	g/dL	12–18	12.9	17.0	<0.0001
Hematocrit	%	37–55	37.0	49.9	<0.0001 ^3^
Leukocytes	×10^3^ cells/mm^3^	6.0–17.0	10.8	10.6	0.84
Neutrophils	×10^3^ cells/mm^3^	3–12	6.5	6.3	0.55
Lymphocytes	×10^3^ cells/mm^3^	1.0–4.8	2.2	2.5	0.60
Eosinophils	×10^3^ cells/mm^3^	0.1–0.9	0.38	0.43	0.50
Total plasmatic proteins	g/L	60–78	79	70	0.0015

^1^ According to the clinical laboratory and also established elsewhere [15,16]. ^2^ Comparison using Mann–Whitney test. ^3^ Non-parametric unpaired *t*-test with Welch correction.

**Table 3:** Comparison of hemogram results between filaria cases and controls.

	Anemia		Bivariate Analysis	
Variable	Positive	Negative	Odds Ratio (95% CI)	*p*-Value
*A. reconditum* ^1^				<0.0001
Positive (n = 49)	30	19	16.3 (5.0–53.1)	
Negative (n = 43)	4	39	1	

^1^ Confirmed by PCR–RFLP. CI: confidence intervals.

## Data Availability

Not applicable.

## Supplementary Materials

The following supporting information can be downloaded at: https://www.mdpi.com/article/10.3390/pathogens11121434/s1, Figure S1: Georeferentiation of Canine filariasis in Cali during 2018–2019, highlighting some environmental features such as surface temperature, rivers, and wetlands; Table S1: Data from Filaria cases and controls from the study.
