# Supplementary figures and images for: First Report of Acanthocheilonema reconditum Outbreak in Canines with Clinical Signs of Anemia from Southwestern Colombia
Source: Pathogens. 2022 Nov 28;11(12):1434. doi: 10.3390/pathogens11121434 (PMC9788614; doi:10.3390/pathogens11121434)

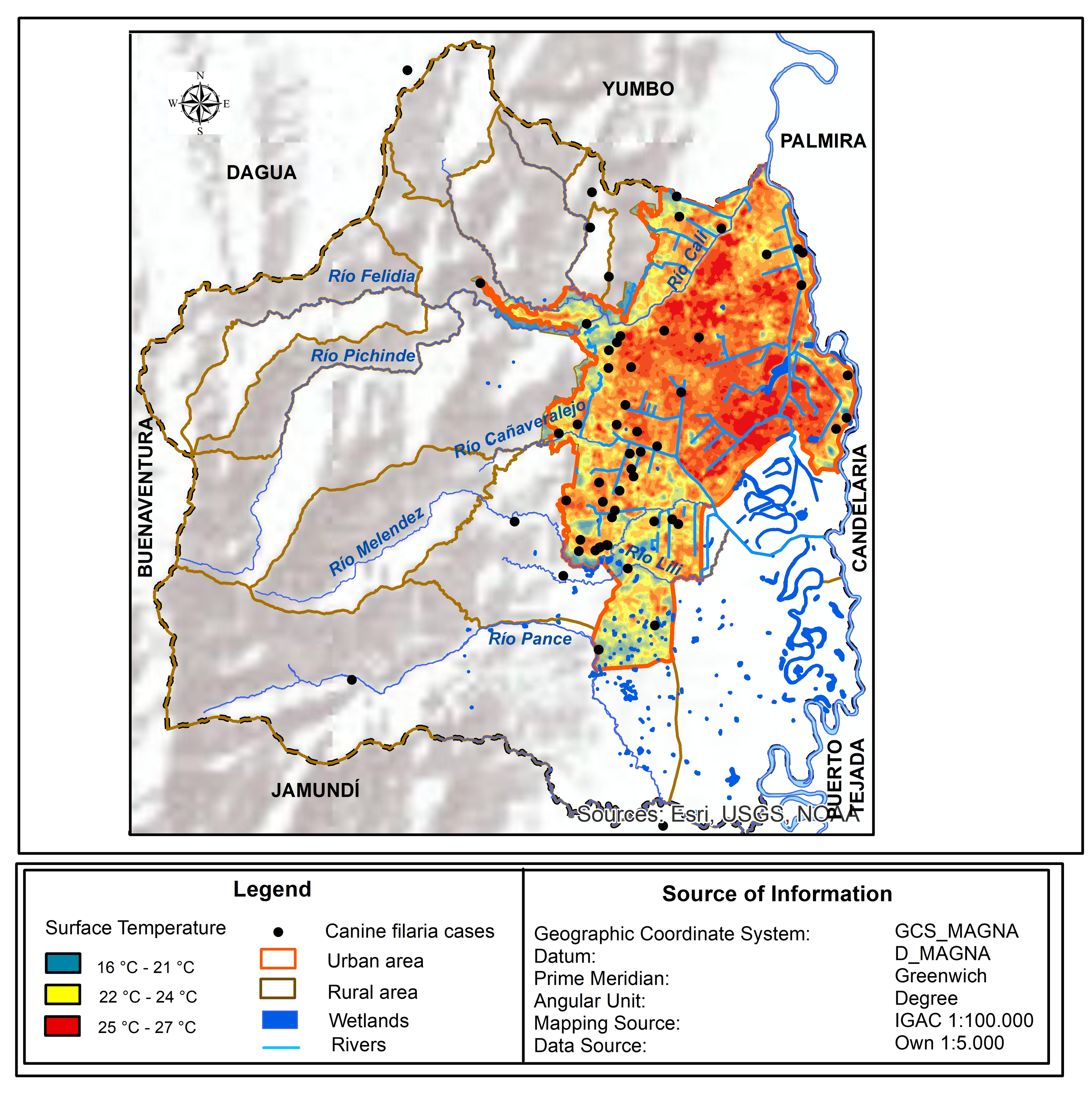

Supplement: Supplementary file 1 [file pathogens-11-01434-s001.zip › Supplementary Figure S1.tif]
